# Growth, Carcass Traits, Blood Chemistry and Gut Microbiota in Broiler Chickens Fed Diets Enriched With Garden Cress Seed Powder as a Natural Growth Enhancer

**DOI:** 10.1002/vms3.70397

**Published:** 2025-05-14

**Authors:** Mohamed H. Negm, Ahmed K. Aldhalmi, Elwy A. Ashour, Laila A. Mohamed, Mahmoud Kamal, Aya Rashad, Mohammad M. H. Khan, Mohamed E. Abd El‐Hack, Ayman A. Swelum

**Affiliations:** ^1^ Poultry Department Faculty of Agriculture Zagazig University Zagazig Egypt; ^2^ College of Pharmacy Al‐Mustaqbal University Babylon Iraq; ^3^ Laboratory of Gastrointestinal Microbiology National Center for International Research on Animal Gut Nutrition Nanjing Agricultural University Nanjing China; ^4^ Department of Animal Nutrition Faculty of Veterinary Animal and Biomedical Sciences Sylhet Agricultural University Sylhet Bangladesh; ^5^ Department of Industrial Pharmacy College of Pharmaceutical Sciences and Drug Manufacturing Misr University for Science and Technology (MUST) Giza Egypt; ^6^ Department of Animal Production College of Food and Agriculture Sciences King Saud University Riyadh Saudi Arabia

**Keywords:** blood, broilers, carcass, cress seed, growth, healthy product, microbiota

## Abstract

This study investigated the effects of garden cress seed powder (GCSP) as a natural growth promoter and antioxidant agent in broiler diets, focusing on growth performance, carcass characteristics, microbial load and blood traits. A total of 210 1‐day‐old Arbor Acres broiler chicks were assigned to 3 experimental groups in a completely randomized design. Each group consisted of 7 replicates with 10 unsexed chicks per replicate. The dietary treatments included: (A) a basal diet without additives (control); (GCSP 1) a basal diet supplemented with 1 g GCSP/kg diet and (GCSP 2) a basal diet supplemented with 2 g GCSP/kg diet. The results revealed no significant differences in body weight (BW), BW gain (BWG), feed intake (FI) and feed conversion ratio (FCR) between the GCSP‐treated groups and the control. However, numerically, the GCSP‐supplemented groups exhibited improved BW, BWG and FCR compared to the control. Carcass traits remained largely unaffected, except for dressing percentage, carcass yield, thigh proportion and abdominal fat percentage, where significant differences were observed. Blood biochemical analysis showed a reduction in total protein, albumin and globulin levels in GCSP‐fed groups, whereas high‐density lipoprotein (HDL) levels increased and low‐density lipoprotein (LDL) and very LDL (VLDL) levels decreased, though these changes were not statistically significant. Immunological and antioxidative responses improved, as evidenced by elevated immunoglobulin Y (IgY), immunoglobulin M (IgM) and superoxide dismutase (SOD) levels, alongside reduced malondialdehyde (MDA) levels in the GCSP‐fed groups compared to the control. In conclusion, dietary supplementation with GCSP enhanced broiler immunity and antioxidative status, promoting increased IgY, IgM and SOD levels while reducing MDA levels. These findings highlight GCSP as a potential natural additive to improve broiler health and contribute to the production of healthier poultry products for consumers.

## Graphical abstract

The present study aimed to show the influence of garden cress seed powder (GCSP) as a natural growth promoter and antioxidant agent in broiler diets on growth performance, carcass characteristics, microbial load and blood traits. Three experimental groups received 210 1‐day‐old broiler chicks (Arbor Acres) in an experiment with a completely randomized design. The groups were separated into 7 duplicates, each containing 10 unsexed chicks. The treatments were as follows: A: without additive (basal diet); GCSP 1 and GCSP 2: basal diet +1 and 2 g garden cress seed powder/kg diet. The results indicated no significant differences among the GCSP treatments in BW, BWG, FI and FCR. However, numerically, the groups fed 2.0 and 1.0 g of GCSP/kg diet showed higher BW, BWG and FCR than the control group. Our results show no significant differences in carcass traits except for dressing, carcass, thigh and abdominal fat proportions. Our findings found that incorporating garden cress seed powder into broiler chick diets reduced blood protein, albumin and globulin levels, increased HDL levels and decreased LDL and VLDL levels in broiler chicks, with no significant changes observed. The results indicate an improvement in immunity and antioxidative blood, with a rise in IgY, IgM and SOD levels and a decrease in MDA levels in the groups fed 2.0 and 1.0 g of GCSP/kg diet, in comparison to the control. In conclusion, using garden cress seed powder in broiler chicks enhanced immunity and antioxidative blood, increasing IgY, IgM and SOD levels while decreasing MDA levels and having a healthy product for end users.

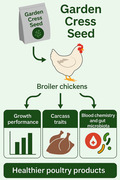

**Abbreviations**
AGP: antibiotic growth promoters
ALT: alanine aminotransferase
AST: aspartate aminotransferase
DBWG: daily weight growth
FCR: feed conversion ratio
FI: feed intake
GCSP: garden cress seed powder
HDL: high‐density lipoprotein
LBW: live body weight
LDL: low‐density lipoprotein
MDA: malondialdehyde
SCFA: short‐chain fatty acid
SOD: superoxide dismutase
TBC: total bacterial count
TC: cholesterol
TG: triglyceride

## 1 Introduction

Feed additives play a crucial role in the poultry industry, which serves as a major source of animal protein worldwide. Feed costs account for approximately 60%–70% of total chicken production expenses, making the optimal utilization of feed a critical factor for economic sustainability (Llanes and Ramirez 2022; Abd El‐Hack, Majrashi et al. 2024; Mohamed et al. 2024). The incorporation of organic feed supplements, including natural growth stimulants and antimicrobials, has been shown to improve growth efficiency, enhance carcass characteristics and support overall health in poultry and other farm animals (Abd El‐Hack, Kamal, Alazragi et al. 2023; Kamal, Aljahdali et al. 2023, Kamal, Kishk et al. 2023; El‐Ratel et al. 2024).

The ban on antibiotic growth promoters (AGP) in poultry diets by the European Union in 2006 due to the rising concern over antimicrobial resistance, the proliferation of foodborne pathogens like *Salmonella* and *Campylobacter*, and the increased risk of human infections have intensified the search for alternative growth enhancers (Chantziaras et al. 2014; Abd El‐Hack, Kamal, Alqhtani et al. 2023). Consequently, researchers have been actively exploring natural feed additives that exhibit comparable mechanisms of action to AGPs while simultaneously improving poultry health, production efficiency and the safety of poultry products (Abd El‐Hack, Ashour et al. 2024; Yang et al. 2024).

Modern intensive poultry production systems expose birds to various environmental and physiological stressors, including oxidative stress, which negatively impacts health, immune function and meat quality (Sola‐Ojo et al. 2019). Although synthetic antioxidants, such as butylated hydroxytoluene (BHT), butylated hydroxyanisole (BHA) and propyl gallate, have been used to mitigate oxidative stress and delay lipid oxidation in feeds, their safety has been questioned due to their potential carcinogenic effects (Felter et al. 2021). In contrast, natural antioxidants derived from plant sources have gained prominence as safe, effective and sustainable alternatives, with many extracted from agricultural and forest residues (Tsiplakou et al. 2021).

Among natural additives, *Lepidium sativum*, commonly known as garden cress, has emerged as a promising candidate due to its rich phytochemical profile and various biological activities. This annual herbaceous plant belonging to the Brassicaceae family is widely consumed in Europe and other regions as a salad ingredient and seasoning (Diwakar et al. 2010). The seeds contain an array of bioactive compounds, including lepidine (a diuretic), imidazole (a blood pressure regulator), glucosinolates (anticancer agents), flavonoids (antioxidants) and semi‐lepidinosides (anti‐inflammatory and anti‐asthmatic properties) (Tiwari et al. 2013; Jain and Grover 2018). Moreover, garden cress seed (GCS) oil is rich in essential fatty acids, such as oleic and linolenic acids, and is characterized by a high lipid content and abundant tocopherols, which contribute to its antioxidant potential and nutritional value (Diwakar et al. 2008).

The unique morphological and sensory attributes of GCSs include their angular, reddish appearance, slight curvature at one end, uniform texture, strong taste and absence of odour (Amawi and Aljamal 2012). The seeds’ diminutive and spherical morphology has also been well‐documented (Gupta and Choudhary 2020). Given their diverse bioactive composition, GCSs have been extensively studied for their potential as a functional feed additive with various health benefits.

The application of GCS powder (GCSP) as a natural feed additive in poultry diets presents multiple advantages, including improved growth performance, enhanced feed conversion ratio (FCR), better carcass quality and a strengthened immune response. Additionally, its use could mitigate the risks associated with synthetic antimicrobial compounds and synthetic antioxidants. Given these benefits, this study aimed to evaluate the effects of incorporating GCSP into broiler diets as a natural growth promoter and antioxidant. Specifically, we assessed its impact on growth efficiency, carcass traits, microbial load and blood biochemical parameters to explore its potential role in producing healthier poultry products.

## 2 Materials and Methods

### 2.1 Birds, Diets and Experimental Design

A completely randomized design experiment was conducted using 210 1‐day‐old Arbor Acres broiler chicks, which were evenly distributed into 3 experimental groups with comparable initial body weight (BW). Each group was further divided into 7 subgroups, with each subgroup consisting of 10 unsexed chicks. The experiment lasted for 31 days, following standard poultry management practices.

The diets were formulated to meet the nutritional requirements established by the National Research Council (NRC 1994). Cress seed powder was incorporated into the diet at specified concentrations before the pelleting process. The mixed feed ingredients underwent pelleting at a temperature of 81°C for 31 s, with a relative humidity of approximately 17.5%. After pelleting, the feed was cooled and subsequently crushed to a particle diameter of less than 1 mm for the starter phase (Days 1–21). The transition from the starter to the finisher feed occurred gradually over 3 days, ensuring a smooth dietary shift before the birds were exclusively fed the finisher's diet until the conclusion of the experiment at 31 days of age (Table 1).

**TABLE 1:** Composition and analytical calculation of the foundational diet.

Items	Starter (1–21 days)	Finisher (22–31 days)
Ingredients (%)		
Soybean meal (44%)	34.00	29.5
Yellow corn	55.05	56.62
Corn germ (60%)	5.50	5.50
Dicalcium phosphate	1.7	1.75
Limestone	1.2	1.23
Premix^a^	0.30	0.30
Salt	0.30	0.30
l‐lysine	0.19	0.0
dl‐methionine	0.12	0.0
Soybean oil	1.64	4.8
Calculated analysis^b^		
CP %	23.3	21.20
ME (kcal/kg)	2954	3153
Calcium %	0.99	1.0
Available phosphorus %	0.45	0.45
M + C	0.9	0.72
Lysine	1.3	1.00
Crude fibre	3.66	3.38
Ether Extract	2.5	2.53
Potassium %	0.54	0.51

Abbreviations: CP, crude protein; ME, metabolizable energy.

^a^ Minerals and vitamins premix manufactured by Multi Vita Animal Nutrition (Tenth of Ramadan City, Sharkia Governorate, Egypt) provides vitamin A 12,000 IU, vitamin D3 2500 IU, vitamin E 20 mg, vitamin K3 2 mg, vitamin B1 2 mg, vitamin B2 5 mg, vitamin B6 2 mg, vitamin B12 0.05 µg, niacin 30 mg, biotin 0.05 µg, folic acid 1 mg, pantothenic acid 10 mg, manganese 60 mg, zinc 50 mg, iron 40 mg, copper 10 mg, iodine 0.6 mg, selenium 0.3 mg/1 kg diet. dl‐methionine (manufactured by Evonik Industries, Essen, Germany) contains 99% methionine. Lysine = lysine hydrochloride (Evonik Industries) and contains 70% lysine.

^b^ Calculated according to NRC (1994).

The experimental groups consisted of

- Control Group (A): Basal diet without additives.
- GCSP1 Group: Basal diet supplemented with 1 g GCSP per kg of feed.
- GCSP2 Group: Basal diet supplemented with 2 g GCSP per kg of feed.

All chicks were reared under identical environmental, managerial and hygienic conditions to ensure experimental consistency. The birds were housed in conventional enclosures with dimensions of 100 × 100 × 50 cm^3^ and were provided ad libitum access to feed and water. Throughout the study period, the birds were subjected to a controlled photoperiod of 23 h of light and 1 h of darkness per day to optimize growth performance.

Environmental controls, including temperature, ventilation and humidity, were maintained within optimal ranges to minimize stress and ensure uniform development across all groups. Routine health monitoring was performed, and any deviations in behaviour, feed intake (FI) or general well‐being were documented to ensure accurate interpretation of the results. These controlled conditions facilitated a precise comparison between the control and treatment groups, allowing for a reliable assessment of the impact of GCSP on broiler growth performance, carcass traits and physiological responses.

### 2.2 Analysis of GCSP

The total phenolic content of GCSP was determined using a UV–Vis spectrophotometer through a reaction with a mixture of extract solution, Folin–Ciocalteu reagent and water. The phenolic concentration was quantified in gallic acid equivalents (GAE) based on a standard gallic acid calibration curve (Lister and Wilson 2001). To assess flavonoid content, a 5% sodium nitrite solution was added to the extract solution, followed by the sequential addition of aluminium chloride and sodium hydroxide. The resulting mixture was diluted, and its flavonoid concentration was determined by measuring optical density at 510 nm (Huang et al. 2010). The antioxidant activity of GCSP was evaluated using the 2,2‐diphenyl‐1‐picrylhydrazyl (DPPH) radical‐scavenging assay. The DPPH solution was prepared in ethanol and added to the extract, with absorbance readings taken at 517 nm against control samples, following the protocol described by Nounah et al. (2017). Table 2 presents the chemical composition and antioxidant activity profile of GCSP, highlighting its bioactive compounds and their potential health benefits.

**TABLE 2:** Chemical composition of antioxidant capacity of garden cress seeds powder.

Ingredient	Content
TPCs (mg GAE/g extract)	22.33
TFs (mg QE/g extract)	2.36
DPPH 100%	19.66
DPPH 200%	34.33
DPPH 400%	48.33
DPPH 800%	85.66

Abbreviations: DPPH, 2,2‐diphenyl‐1‐picrylhydrazyl; QE, quercetin equivalents; TFs, total flavonoids; TPCs, total phenolic compounds. Antioxidant activity.

### 2.3 Collecting Data

The chicks were individually weighed each week to determine their live BW (LBW) and daily weight growth (DBWG). The mean daily FI and daily FCR were computed.

### 2.4 Carcass Traits

We randomly selected 7 birds from each group, around the mean of the treatment, for carcass evaluations at 31 days of age. After a night of fasting, we manually weighed and slaughtered these birds. The carcass weight and the weight of all other edible portions were measured.

### 2.5 Biochemical Examination of the Blood

After an overnight fasting period, we collected blood specimens by euthanizing seven birds from each group. The blood was obtained in heparin‐free tubes and subsequently subjected to centrifugation at a speed of 5000 rpm for 15 min at a temperature of 4°C. The resultant serum was stored at −20°C until it underwent biochemical analysis. The samples of serum were used to identify ‘serum alanine aminotransferase (ALT) and aspartate aminotransferase (AST) activities, as well as the measurement of total protein, albumin, total cholesterol, triglycerides (TG), low‐density lipoprotein (LDL) and high‐density lipoprotein (HDL)’ levels. Furthermore, following the manufacturer's instructions for kits that measure creatinine levels and immunological responses, such as immunoglobulin Y (IgY) and immunoglobulin M (IgM), is important. The levels of malondialdehyde (MDA) and superoxide dismutase (SOD), which are markers of oxidative state, were measured using the methods described in our earlier work (Abd El‐Hack et al. 2017). Personal protective equipment was used appropriately while collecting and organizing the samples.

### 2.6 Caecal Microbiota

The cecum of the slaughtered birds was subsequently taken for microbiological investigation. We sterilized 1.0 g of cecum from seven hens per group to measure total bacterial count (TBC), coliforms, *Escherichia coli*, *Salmonella*, *Clostridium*, *Lactobacilli*, *Lactococci* and *Bacillus*. The collected samples were stored in cryovials at −80°C until analysis. Microbes were cultivated on a small scale using a previously described method (Sieuwerts et al. 2008).

### 2.7 Statistical Analysis

The data underwent ANOVA using a completely randomized design. SAS (2001) generalized linear model techniques were used for this analysis. The distinctions between means were ascertained utilizing the post hoc Tukey's test. Significance statistics were determined using a threshold of (*p* < 0.05) except further specified. The subsequent statistical model was employed:

The equation *Y_ij_* = *μ* + *T_i_* + *e_ij_* represents the relationship among the measured value of the relevant treatment (*Y_ij_*), the observed mean for the relevant treatment (*μ*), the treatment impact (*T_i_*) and the error associated with individual observation (*e_ij_*).

## 3 Results

### 3.1 Total Phenols, Flavonoids and Antioxidant Activity

The total phenolic and flavonoid contents were quantified using gallic acid and quercetin as reference standards, respectively. These bioactive compounds play a crucial role in plant metabolism and exhibit significant antioxidant properties. As presented in Table 2, the total phenolic content was determined to be 22.33 mg GAE/g of extract, highlighting the strong presence of polyphenolic compounds. Similarly, the total flavonoid content was found to be 2.36 mg quercetin equivalents (QE)/g of extract, indicating a moderate concentration of flavonoid compounds known for their free radical‐scavenging capabilities.

To evaluate the antioxidant potential of GCSP, the widely recognized DPPH assay was employed. This method assesses the ability of the extract to donate hydrogen atoms or electrons to neutralize DPPH radicals, thereby measuring its free radical‐scavenging activity. The antioxidant activity was determined by monitoring the reduction of DPPH radicals at different concentrations, expressed as the percentage of inhibition at specified doses.

The concentration required to inhibit 100%, 200%, 400% and 800% of DPPH radical activity is referred to as IC100‐800 for DPPH scavenging activity. The extract exhibited notable DPPH radical‐scavenging activities, with inhibition percentages of 19.66% at DPPH 100, 34.33% at DPPH 200, 48.33% at DPPH 400 and 85.66% at DPPH 800. These results demonstrate a clear dose‐dependent increase in antioxidant activity, suggesting that higher concentrations of the extract provide greater protective effects against oxidative stress.

The high radical‐scavenging ability of GCSP can be attributed to its rich composition of bioactive compounds, including phenolic acids, flavonoids and other secondary metabolites. These compounds play a crucial role in mitigating oxidative damage by neutralizing reactive oxygen species (ROS), thereby contributing to cellular protection and overall health benefits. The findings reinforce the potential of GCSP as a natural source of antioxidants, which may be utilized in functional food formulations and nutraceutical applications aimed at enhancing human and animal health.

### 3.2 Growth Performance

Tables 3, 4, 5, 6 present the effects of GCSP on BW, BW gain (BWG), FI and FCR, respectively. We recorded no significant differences among the GCSP treatments in BW, BWG and FCR. We observed higher BW, BWG and FCR in the treatments fed 2.0 and 1.0 GCSP (g/kg diet) in contrast to the control. The FI showed notable variations, with the 2.0 GCSP (g/kg diet) group having the highest FI, followed by the 1.0 GCSP (g/kg diet) group and the control.

**TABLE 3:** Live body weight of broiler chickens when administered varying doses of dietary GCSP supplementation.

Items	LBW (g)					
	Initial	7 D	14 D	21 D	28 D	31 D
GCSP (g/kg diet)						
0.0	45.29	169.81	412.50	923.17	1597.98	1889.15
1.0	45.21	168.85	408.85	928.75	1606.35	1924.95
2.0	45.10	173.65	413.66	927.41	1600.09	1925.92
SEM	0.55	5.31	6.78	12.94	13.99	39.85
*p* value	0.935	0.567	0.723	0.890	0.798	0.498

Abbreviations: D, days; GCSP, garden cress seed powder; LBW, live body weight; SEM, standard error mean.

**TABLE 4:** Influence of nutritional supplementation with GCSP on BWG in broiler chickens.

Items	BWG (g/day)					
	1–7 D	8–14 D	15–21 D	22–28 D	29–31 D	1–31 D
GCSP (g/kg diet)						
0.0	17.68	34.59	72.92	96.43	97.06	63.74
1.0	17.66	34.29	74.27	96.80	106.20	65.84
2.0	18.37	34.29	73.39	96.10	108.61	66.15
SEM	0.70	0.40	2.65	2.24	11.22	3.41
*p* value	0.487	0.452	0.862	0.946	0.472	0.055

Abbreviations: BWG, body weight gain; D, days; GCSP, garden cress seed powder; SEM, standard error mean.

**TABLE 5:** Effect of nutritional supplementation with GCSP on FI in broiler chickens.

	FI (g/day)					
Items	1–7 D	8–14 D	15–21 D	22–28 D	29–31 D	1–31 D
GCSP (g/kg diet)						
0.0	28.14	52.93	86.11	126.23	129.11^b^	84.50^c^
1.0	28.34	51.07	86.81	123.71	149.10^a^	87.81^b^
2.0	29.35	51.95	87.23	124.26	149.94^a^	88.54^a^
SEM	1.29	1.32	1.91	4.15	10.97	1.93
*p* value	0.487	0.231	0.795	0.766	0.002	>0.001

*Note*: Different letters (a–c) within one column are significantly different (*p* < 0.05).

Abbreviations: D, days; FI, feed intake; GCSP, garden cress seed powder; SEM, standard error mean.

**TABLE 6:** Impact of dietary GCSP supplementation on FCR in broilers.

Items	FCR (g feed/g gain)					
	1–7 D	8–14 D	15–21 D	22–28 D	29–31 D	1–31 D
GCSP (g/kg diet)						
0.0	1.57	1.54	1.19	1.32	1.33	1.39
1.0	1.60	1.49	1.17	1.28	1.42	1.39
2.0	1.60	1.52	1.19	1.29	1.39	1.40
SEM	0.09	0.03	0.02	0.02	0.14	0.03
*p* value	0.967	0.252	0.366	0.291	0.796	0.766

Abbreviations: D, days; FCR, feed conversion ratio; GCSP, garden cress seed powder; SEM, standard error mean.

### 3.3 Carcass Characteristic

Table 7 illustrates the impacts of GCSP on broiler chickens’ organ weights (carcass, breast, thigh, liver, heart, gizzard and giblets) and carcass traits on their BW. An examination of the variance did not reveal any significant variances in treatments for any of the carcass traits studied, except for the proportion of dressing, carcass, thigh and abdominal fat. The proportion of these organs was higher in the control, followed by the 1.0 GCSP (g/kg diet) group and then the 2.0 GCSP (g/kg diet) group.

**TABLE 7:** Effect of dietary GCSP supplementation on carcass characteristics in broilers.

	Relative to pre‐slaughter weight, %								
Items	Dressing	Carcass	Breast	Thigh	Liver	Heart	Gizzard	Giblets	Abdominal fat
GCSP (g/kg diet)									
0.0	80.44^a^	75.95^a^	33.43	29.29^a^	2.27	0.44	1.78	4.49	0.67^a^
1.0	77.39^b^	73.03^b^	33.06	28.00^b^	2.38	0.46	1.51	4.35	0.52^b^
2.0	77.22^b^	72.43^c^	33.13	26.31^c^	2.77	0.45	1.56	4.79	0.32^c^
SEM	1.73	1.79	0.52	1.52	0.36	0.03	0.17	0.37	0.11
*p* value	0.005	0.005	0.713	0.005	0.218	0.796	0.087	0.397	>0.001

*Note*: Different letters (a–c) within one column are significantly different (*p *< 0.05).

Abbreviations: GCSP, garden cress seed powder; SEM, standard error mean.

### 3.4 Haematological and Biochemical Traits of Blood

Table 8 summarizes the impacts of incorporating GCSP as a substance added to broiler chicks’ feed biochemical analysis of blood. The analysis of variance revealed no statistically substantial changes among the treatments in terms of total protein, albumin and globulin. The diet, which contained 2.0 and 1.0 g of GCSP/kg, resulted in decreased levels of ALT and AST in the birds. Furthermore, adding 2.0 and 1.0 g of GCSP/kg of diet increased HDL levels while significantly reducing LDL, very LDL (VLDL) and creatinine levels (*p* < 0.05).

**TABLE 8:** Blood indices of broilers fed different levels of dietary GCSP supplementation.

Items	TP (g/dL)	ALB (g/dL)	GLOB (g/dL)		A/G ratio	ALT (U/L)	AST (U/L)	CREAT (mg/dL)	TC (mg/dL)	TG (mg/dL)	HDL (mg/dL)	LDL (mg/dL)	VLDL (mg/dL)
GCSP (g/kg diet)													
0.0	5.21	2.62		2.59	1.02	20.30^a^	144.02^a^	0.69^a^	137.96	99.03	44.19^c^	70.41^a^	19.80^a^
1.0	4.34	2.70		1.64	1.93	19.51^b^	134.80^b^	0.65^b^	132.62	98.44	45.04^b^	67.88^b^	18.68^a^
2.0	4.34	2.45		1.89	1.34	8.41^c^	74.64^c^	0.34^c^	133.52	82.98	47.74^a^	47.91^c^	16.59^b^
SEM	0.57	0.18		0.60	0.70	5.01^d^	34.18	0.19	22.53	14.90	3.03	18.60	2.98
*p* value	0.072	0.232		0.118	0.306	0.010	0.001	0.010	0.264	0.373	0.034	0.032	0.034

*Note*: Different letters (a–c) within one column differ significantly (*p *< 0.05).

Abbreviations: ALB, albumen; ALT, alanine transaminase; AST, aspartate aminotransferase; CREAT, creatinine; GCSP, garden cress seed powder; GLOB, globulin; HDL, high‐density lipoprotein; LDL, low‐density lipoprotein; SEM, standard error mean; TC, total cholesterol; TG, triglycerides; TP, total protein; VLDL, very low‐density lipoprotein.

### 3.5 Immunity and Antioxidative

Table 9 summarizes the effects of incorporating GCSP as a feed additive on broiler chicks’ immunity and antioxidative blood analysis. The analysis of variance revealed statistically substantial variations among the treatments concerning IgY, IgM, SOD and MDA. The diet, which contained 2.0 and 1.0 g of GCSP/kg diet, resulted in increased IgY, IgM and SOD while significantly decreasing MDA levels (*p* < 0.05) in contrast with the control.

**TABLE 9:** The immunity and antioxidative parameters of broilers were fed at different levels of dietary GCSP supplementation.

Items	IgY (ng/mL)	IgM (ng/mL)	SOD (U/mL)	MDA (nmol/mL)
GCSP (g/kg diet)				
0.0	223.42^c^	318.89^c^	61.69^c^	11.86^a^
1.0	459.20^a^	481.15^b^	144.41^b^	3.08^c^
2.0	371.30^b^	701.96^a^	144.81^a^	5.90^b^
SEM	111.97	168.22	42.10	3.94
*p* value	>0.001	>0.001	>0.001	>0.001

*Note*: Different letters (a–c) within one column differ significantly (*p *< 0.05).

Abbreviations: GCSP, garden cress seed powder; IgM, immunoglobulin A; IgY, immunoglobulin Y; MDA, malondialdehyde; SEM, standard error mean; SOD, superoxide dismutase.

### 3.6 Caecal Microbiota

**FIGURE 1:**
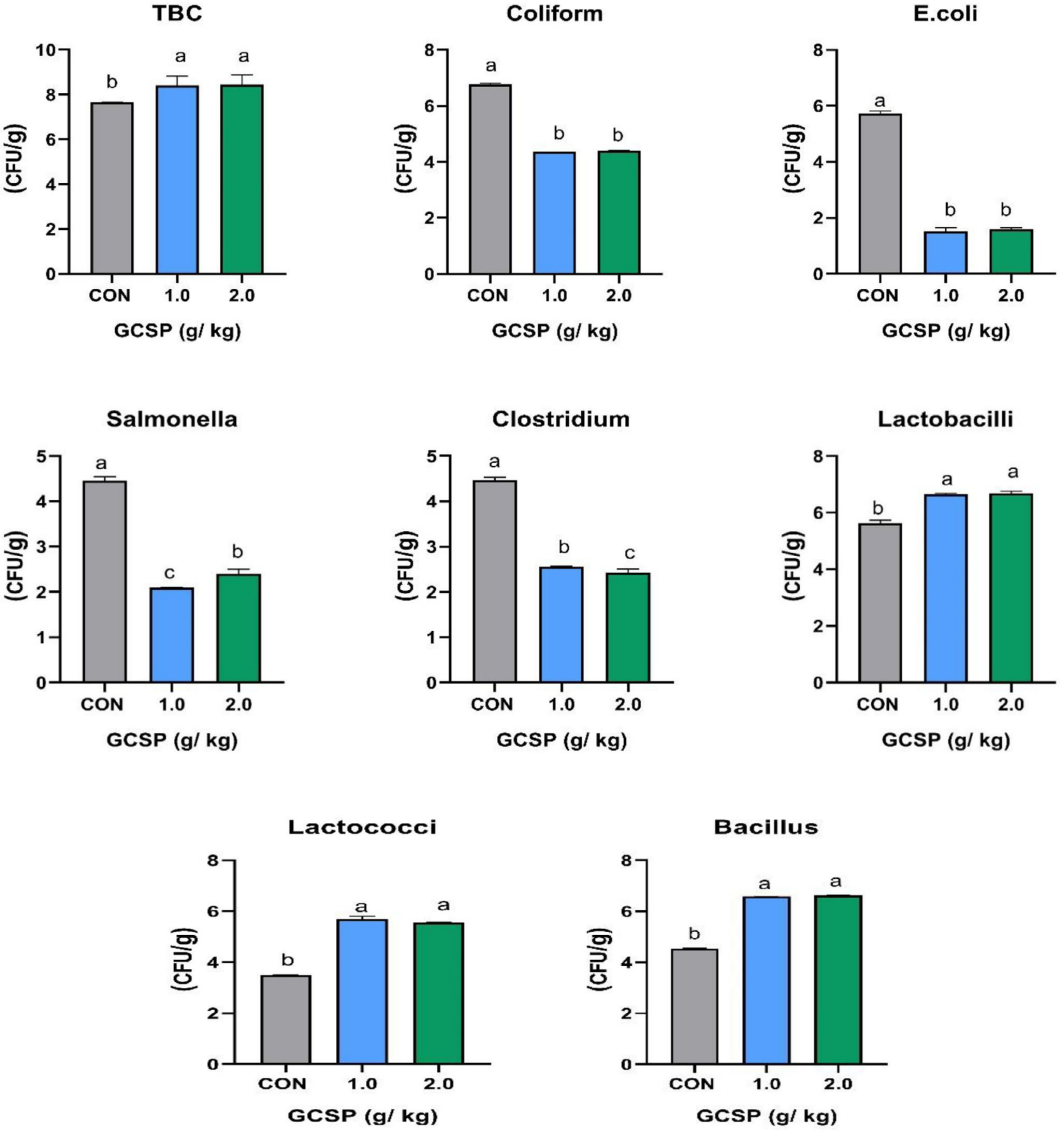
Effect of diets GCSP treatments on the microbial count of broilers cecum. *E. coli*, *Escherichia coli*; GCSP, garden cress seed powder; TBC, total bacterial count.

Figure 1 shows the microbial counts for the caecal inclusions of groups that were given different amounts of GCSP and a control group. When compared to other treatments, the group that was given GCSP at doses of 1.0 and 2.0 g had much lower counts of coliform, *E. coli*, *Salmonella* and *Clostridium* (*p *< 0.05). The results also showed that the groups that got GCSP at 1.0 and 2.0 g had higher amounts of *Lactobacilli*, *Lactococci* and *Bacillus*.

## 4 Discussion

Garden cress (*L. sativum*) has been traditionally utilized in various cultures for its medicinal and nutritional properties. Both its seeds and leaves have been employed as an expectorant, diuretic, galactagogue, stimulant and poultice, and they have been associated with lowering blood pressure (Maghrani et al. 2005). These health benefits are largely attributed to its rich biochemical profile, including essential fatty acids (notably oleic acid at 30.6% and linolenic acid at 29.3%), a significant fat content ranging between 18% and 24% and the presence of bioactive compounds such as tocopherols, lignans and antioxidants (Diwakar et al. 2008). The high nutrient density of GCSs makes them an attractive dietary component for enhancing animal nutrition and growth performance.

Several studies have demonstrated that incorporating GCS into poultry diets can yield significant improvements in performance metrics such as BW, BW gain (BWG), FI and FCR. Hassan and El Shoukary (2019) observed that dietary supplementation with GCSs enhanced BW, BWG and FI in broiler chickens. Similarly, Shawle et al. (2016) reported that broilers fed diets containing 1.5% cress seed exhibited increased BWG and FI relative to the control group. This effect can be attributed to the high nutritional value of cress seed, which enhances the palatability of feed and facilitates efficient nutrient absorption. Further supporting this, Frankič et al. (2009) and Azene et al. (2022) reported that the inclusion of cress seed in animal feed positively influenced FI, improved production efficiency and increased the European broiler index. Additionally, it was found to affect ingestive behaviour, likely due to its palatable nature and the bioavailability of essential nutrients.

Interestingly, a study by Weeks et al. (2000) suggested that although increased BW can be a positive indicator of dietary efficiency, it might also lead to reduced walking activity in birds. This aligns with the findings of Shawle et al. (2016), who demonstrated that adding 1.5% cress seed to broiler diets significantly improved FCR. The efficiency of feed utilization, as evidenced by improvements in FCR and FI, underscores the potential of GCS as a functional feed ingredient. Taghipour et al. (2021) further emphasized the role of cress seed in promoting weight gain due to its high content of *n*‐3 polyunsaturated fatty acids (PUFAs), tocopherols, lignans and antioxidants, which contribute to overall growth performance and metabolic efficiency. Despite the numerous positive findings, some studies have reported neutral effects regarding FI and conversion ratios. Rashnou et al. (2023) found that supplementing quail diets with 50 and 100 g/kg of garden cress and purslane did not significantly alter FI and FCR. Similarly, Moazedian and Saemi (2018) observed no significant changes in FI when broilers were fed diets containing garden cress at levels of up to 2% and 25%. Evaris et al. (2015) suggested that variations in FI among juvenile birds could be attributed to their ability to self‐regulate their FI based on the energy density of their diet. These findings indicate that although GCS can be a valuable dietary supplement, its effects may vary based on factors such as dietary composition, species and physiological adaptations of the animals.

Adding GCS improved the FCR in some ways. It was discovered that cress seeds, which are full of enzymes, help break down complex carbohydrates, proteins and fats in the gut. This makes digestion and absorption of essential nutrients better, which could improve the FCR (Tufail et al. 2024). In addition, GCS may change the makeup of the gut microbiome, which could lead to better nutrient fermentation, short‐chain fatty acid (SCFA) production and digestive health in general (Taghipour et al. 2021). Moreover, GCS, rich in protein, is a valuable supplement for animal diets, ensuring a balanced amino acid profile for efficient protein synthesis and growth (Hassan and El Shoukary 2019). GCSs’ effect on feed conversion rate depends on gut health and nutrient digestibility. Further research is needed to understand intestinal enzyme activity and fibre content, as moderate fibre can promote gut health, whereas optimal addition rates and inclusion levels may vary depending on poultry species.

Beyond its impact on growth performance, GCS has shown promise in enhancing digestibility, immunity and carcass characteristics. Abdelrahman Morshedy et al. (2022) demonstrated that including 3%–4.5% GCS in the diets of growing rabbits improved growth rates, feed utilization, nutrient digestibility and immune response. This suggests that GCS could be considered a functional feed additive to enhance overall health and development in both rabbits and poultry.

In terms of carcass traits, Abdelrahman Morshedy et al. (2022) reported that although GCS supplementation did not significantly impact overall carcass characteristics, it was associated with increased slaughter and skinning weights, reduced kidney fat deposition and notable effects on the lengths of the large intestine and colon. These findings suggest that although the primary benefit of cress seed supplementation lies in improved growth and metabolism, it may also exert subtle effects on body composition and digestive morphology. Shawle et al. (2016) found that incorporating GCS at concentrations of up to 1.5% in broiler diets did not significantly affect liver weight. This suggests that the experimental diets did not introduce toxicological risks that could compromise organ development, further supporting the safety of GCS as a feed additive.

Biochemical blood parameters serve as crucial indicators for assessing an animal's health and physiological state, providing insight into metabolic functions and overall well‐being (Adeyeye et al. 2020; Kamal, Kishk et al. 2023). This study investigated the impact of GCSP supplementation on various blood biochemical markers in broiler chicks, revealing significant modulations in lipid metabolism, protein composition and hepatic function. The findings indicate that incorporating GCSP into broiler diets resulted in a decrease in blood protein, albumin and globulin levels, alongside an improvement in HDL levels and reductions in LDL, VLDL and creatinine levels. These results align with prior studies demonstrating the lipid‐modulating properties of garden cress. Alharbi and Hanan (2017) observed that cress seed supplementation did not alter serum globulin levels in diabetic rats, corroborating the minimal changes seen in the present study. Furthermore, Althnaian (2014) reported that rats consuming cress seed diets did not exhibit significant alterations in serum albumin and calcium levels, supporting the findings that GCSP does not strongly impact these parameters in broiler chicks.

In contrast, Hassan and El Shoukary (2019) documented that cress seed supplementation enhanced immune responses in birds, attributing this effect to increased serum total protein and globulin levels. The presence of essential vitamins such as C and A in cress seeds likely contributed to this immunostimulatory effect. Additionally, Abdelrahman Morshedy et al. (2022) found comparable results, indicating no substantial effects on protein metabolism; however, increasing GCSP levels resulted in decreased cholesterol (TC) and LDL while simultaneously raising HDL, HDL/LDL ratios, TG and VLDL concentrations. These findings are consistent with Chauhan et al. (2012), who reported significant reductions in TC, TG, LDL and VLDL levels, accompanied by an increase in HDL upon dietary inclusion of GCSP. Similarly, Al Hamedan (2010) demonstrated that oral administration of GCSP significantly reduced blood cholesterol and total lipid levels in rats. Amawi and Aljamal (2012) further confirmed these lipid‐lowering effects, showing that a 4‐week administration of *L. sativum* extract (30 mg/kg BW) resulted in substantial reductions (*p* ≤ 0.05) in TC, TG, LDL‐c and VLDL‐c, whereas HDL‐c levels increased significantly. The observed reduction in TG following GCSP supplementation may be attributed to the flavonoid content of medicinal plants like GCSP. Brahmachari (2011) noted that certain flavonoids possess insulin‐mimetic properties, facilitating triglyceride metabolism and reducing lipid accumulation in the bloodstream.

Serum levels of AST and ALT serve as key biomarkers for hepatic function and potential damage (Gu et al. 2021). Elevated concentrations of these enzymes are typically indicative of liver or muscle damage due to physiological stress or illness (Sang et al. 2023). Abdelrahman Morshedy et al. (2022) reported AST values ranging from 193 to 227 IU/L, which remain within the normal physiological range. According to Campbell (2012), plasma AST activity is classified as elevated when it exceeds 275 IU/L, reinforcing that GCSP consumption in this study did not induce hepatic distress. Supporting this observation, Youssef et al. (2014) found that garden cress oil significantly reduced AST and ALT levels in hypercholesterolemic albino rats, particularly at concentrations ranging from 25% to 75%. These findings suggest that garden cress may possess hepatoprotective properties, mitigating liver enzyme elevations typically associated with metabolic disorders.

Garden cress (*L. sativum*) is a nutrient‐dense plant rich in proteins, fatty acids, minerals and vitamins, including kaempferol glucuronide, gallic acid, protocatechuic acid, coumaric acid, caffeic acid, terpenes and glucosinolates. The presence of these bioactive compounds imparts significant medicinal and functional benefits, including antioxidant, thermogenic and antiscorbutic properties (Tufail et al. 2024). The beneficial effects of GCSP on lipid and hepatic parameters can be attributed to its high content of flavonoids, alkaloids and omega‐3 fatty acids. Pérusse et al. (2014) emphasized that omega‐3 fatty acids play a crucial role in cardiovascular health, reducing inflammation, preventing heart attacks and enhancing immune function. Additionally, flavonoids present in GCSP exert hypolipidemic effects by modulating key metabolic pathways, leading to improved lipid profiles and reduced oxidative stress.

GCSs are found to contain 18%–24% fat (Gopalan and Sastri 2004), of which ∼34% of total fatty acids are alpha‐linolenic acid (ALA) (Gokavi et al. 2004). The principal motivation for using GCS in broiler diets is its capacity to enhance the meat's omega‐3 fatty acid content, particularly ALA (18:3*n*‐3). GCSs are a valuable source of ALA. When administered to broilers, a portion of the ALA is metabolized into longer chain omega‐3 fatty acids, specifically eicosapentaenoic acid (EPA, 20:5*n*‐3) and docosahexaenoic acid (DHA, 22:6*n*‐3) (Diwakar et al. 2010). In addition to omega‐3 fatty acids, GCS can alter the total fatty acid profile, potentially impacting the ratios of SFAs, MUFAs and PUFAs (Maheswaraiah et al. 2018). The primary motivation for increasing omega‐3 content in broiler meat is to improve its nutritional value and provide human health benefits. Omega‐3 fatty acids are known to reduce the risk of cardiovascular disease by lowering TG, blood pressure and inflammation (Alagawany et al. 2019). Additionally, omega‐3s have anti‐inflammatory properties that can help with conditions like arthritis (Kostoglou‐Athanassiou et al. 2020). More research is needed to optimize the inclusion rate and form of GCS in broiler diets to maximize omega‐3 enrichment without compromising broiler performance.

Our findings demonstrate that the inclusion of GCS powder in broiler diets leads to significant immunological and antioxidant benefits. Notably, IgY and IgM levels increased, along with elevated SOD activity, whereas MDA levels decreased compared to the control group. This suggests an improved immune response and enhanced oxidative stress defence mechanisms in broiler chicks supplemented with GCS. MDA is a well‐known biomarker for oxidative stress, generated as a byproduct of PUFA peroxidation within cell membranes. Its elevated levels indicate excessive oxidative damage due to free radical activity (Gaweł et al. 2004). By contrast, SOD is a critical antioxidant enzyme that converts superoxide radicals into hydrogen peroxide and molecular oxygen, mitigating cellular oxidative stress (Gurudath et al. 2012). The upregulation of SOD in GCS‐fed broilers underscores its potential role in enhancing the bird's ability to neutralize ROS, thereby reducing cellular damage.

Naturally occurring antibodies in poultry serum play a fundamental role in distinguishing between fast‐ and slow‐growing broilers. By fortifying diets with GCS, significant reductions in cholesterol, TG and certain fatty acids—such as α‐linolenic acid and arachidonic acid—were observed in both liver tissues and serum. This modulation suggests that GCS facilitates improved lipid metabolism and enhances the conversion of dietary linoleic acid into long‐chain PUFAs like EPA and DHA (Diwakar et al. 2010; Dai et al. 2023). The enhanced synthesis of these PUFAs in the liver, heart, serum and brain tissues further supports GCS as a valuable nutritional intervention for optimizing metabolic functions in poultry.

Beyond their physiological effects, GCSs exhibit potent antioxidant properties attributable to their high concentrations of phenolic compounds, phytosterols and tocolytic substances. These bioactive compounds inhibit lipid peroxidation, which is crucial for both biological and industrial applications. The high DPPH free radical‐scavenging activity of GCS also makes it a promising candidate for fortifying food products (Al‐Saad and Al‐Saadi 2021). Several studies have confirmed the superior antioxidant activity of GCS across various plant parts. In a comparative analysis, the whole plant exhibited the highest radical‐scavenging capacity, surpassing that of its leaves and stem (Malar et al. 2014). The presence of coumaric acid in GCSs further amplifies their antioxidant and anti‐inflammatory effects, which could have potential therapeutic applications (Kadam et al. 2018).

Ethanolic extracts of GCS have demonstrated remarkable antioxidant activities across different assays, with significant values recorded for superoxide‐reducing activity, DPPH radical‐scavenging, metal‐chelating capacity and ABTS scavenging ability (Attia et al. 2019). Moreover, Abdelrahman Morshedy et al. (2022) highlighted the immunomodulatory effects of GCS in rabbits, showing a significant increase in SRBC levels, particularly after 14 days of supplementation. Similarly, Qusti et al. (2016) reported that dietary inclusion of GCS led to a substantial rise in immunoglobulin concentrations in human participants, reinforcing the immunostimulatory potential of these seeds. Al‐Saad and Al‐Saadi (2021) further emphasized the role of GCS in disease prevention, attributing these benefits to its potent antioxidant and antiradical properties.

At the molecular level, GCS‐derived antioxidant compounds, including phenolic acids, polyphenols and flavonoids, actively neutralize a variety of ROS, such as hydroxyl radicals, hydroperoxides and lipid peroxyl radicals. They also counteract hydrogen peroxide and superoxide anions, thereby mitigating oxidative stress‐related cellular damage, which is linked to chronic diseases (Sharma 2015). In alignment with these findings, Buso et al. (2020) reported that *L. sativum* seeds possess significant anti‐inflammatory properties, reinforcing their potential in both dietary and medicinal applications.

Dietary supplementation with bioactive compounds, such as those found in GCS, represents a promising strategy for optimizing broiler growth and health (Guo et al. 2022; Ashour, Aldhalmi, Ismail et al. 2025; Ashour, Aldhalmi, Kamal et al. 2025). However, the impact of herbal extracts on broiler performance remains contentious. Although Maes et al. (2019) and El‐Abasy et al. (2025) observed no significant enhancement in growth efficiency following herbal extract supplementation, they also reported no adverse effects, indicating that such interventions are at least safe for poultry use. Notably, water quality remains a critical factor influencing poultry health and performance, often overshadowing dietary modifications.

Given their essential part in intestine development and metabolic balance (Dittoe et al. 2022), intestinal microbes are attracting more and more attention nowadays. In terms of nutrient digestion and absorption, the small intestine, colon and cecum serve somewhat comparable functions. The most crucial distal intestine section of chickens is their cecum, so the intestinal microbe concentration is highest in adult chickens. The health and growth performance of the body are affected by the much bigger quantity of microorganisms than in other intestinal segments in poultry with different microbiota (Yin et al. 2023). Consequently, one should investigate the intestinal microbial diversity of chickens.

GCSs contain dietary fibre, complex carbohydrates and resistant starch, which can be fermented by gut bacteria (Azene et al. 2022). When soaked in water, they form a gelatinous mucilage, which may contribute to prebiotic potential. The specific fibres and carbohydrates in these seeds may favour certain beneficial bacterial species. Garden cress may impact gut microbiome composition, potentially improving nutrient fermentation, SCFA production and overall digestive health through a healthier, balanced gut microbiome (Tufail et al. 2024). SCFA are generated as a result of bacterial fermentation in the cecum. They stimulate cell development and differentiation in the intestine, enhancing intestinal integrity, as well as decreasing the digestive tract pH and limiting the growth of pathogenic microbes (Knudsen et al. 2012). The findings of this investigation indicated a decrease in *E. coli* and a rise in *Lactobacilli, Lactococci* and *Bacillus* bacteria. Wu et al. (2022) said that the concentration of oregano essential oil did not have a big effect on the variety and richness of the caecal microbial colonies of the broiler. This could be brought on by oregano essential oil's antibacterial action against intestinal pathogens, including *E. coli* and *Clostridium perfringens* (Chouhan et al. 2017). The ambient conditions and basic diets of chickens could influence the antimicrobial effects of essential oils (Leyva‐López et al. 2017). Essential oil, a mix of thyme and organic acid, was given to male turkeys. There was no statistically significant difference between the groups in the amount of caecal SCFA production (Mikulski et al. 2008). Although acetic acid rates dropped on the 20th day, a mix of thymol and cinnamaldehyde was found to improve butyrate rates on the 20th and 41st days (Tiihonen et al. 2010). In another investigation, combined plant extract and enzymes raised caecal volatile fatty acid rates on the 22nd day (Cao et al. 2010). Furthermore, the administration of herbal extracts has been shown to beneficially modulate gut microbiota. Meng et al. (2023) found that herbal supplementation significantly increased the populations of beneficial bacterial genera, such as *Lactobacillus* and *Enterococcus*, while reducing *Dysgonomonas* levels in the cecum. *Lactobacillus* and *Enterococcus* are known to produce SCFAs, which play key roles in gut health by reducing inflammation and maintaining intestinal barrier integrity (Hati et al. 2019). Probiotic formulations containing these bacteria have consistently demonstrated efficacy and safety in poultry diets (Zhang et al. 2018). On the other hand, *Dysgonomonas* is recognized as a potential pathogen in poultry. As reported by Rahimi et al. (2011), herbal supplementation effectively shifts the gut microbiota composition, promoting beneficial microbes while suppressing pathogenic bacteria, thereby fostering a healthier intestinal environment. The different results could be because of different herbal elements, such as the type and amount of plant extracts used, the ratio of volatile fatty acids and active ingredients, interactions and ratio composition, the conditions of the breeding and environmental factors.

In summary, our findings reinforce the potential of GCSP as a valuable dietary supplement for improving poultry health. Its multifaceted benefits, ranging from enhanced immune function and lipid metabolism to improved antioxidant defence mechanisms and gut microbiota modulation, position GCS as a promising candidate for poultry nutrition. Further investigations should focus on optimizing dosages and assessing long‐term effects to maximize its application in commercial poultry production.

## 5 Conclusions

The findings of this study highlight the beneficial effects of incorporating GCSP into broiler chick diets, showing significant improvements in immunity and antioxidant status. Supplementation with GCSP resulted in increased levels of IgY, IgM and SOD, alongside a reduction in MDA levels compared to the control group. These changes reflect an enhanced immune response and a decrease in oxidative stress, both of which are vital for maintaining optimal bird health and welfare. On the basis of these results, we recommend including 1.0–2.0 g of GCSP per kg of diet to support better growth performance, stronger immunity and improved antioxidant defences. This supplementation not only promotes overall health but also contributes to the production of healthier poultry meat. Moreover, the inclusion of GCSP reduces reliance on antibiotics, helping to address concerns related to antibiotic resistance in poultry production. In addition, birds supplemented with GCSP exhibited healthier lipid profiles, with lower levels of ‘bad’ cholesterol (e.g., LDL) and higher levels of ‘good’ cholesterol (e.g., HDL). These findings suggest that GCSP can enhance the nutritional quality of broiler meat, making it a healthier option for consumers.

In conclusion, the inclusion of GCSP in broiler diets represents a promising natural strategy to enhance growth performance, immune function, antioxidant status and overall health. This approach offers an effective alternative to AGP, ultimately contributing to the production of high‐quality, health‐promoting poultry products.

## Author Contributions

**Mohamed E. Abd El‐Hack, Elwy A. Ashour** and **Laila A. Mohamed**: Conceptualization and supervision. **Elwy A. Ashour, Mohamed H. Negm, Mahmoud Kamal** and **Aya Rashad**: Methodology and investigation. **Mohamed E. Abd El‐Hack, Mohammad M. H. Khan, Laila A. Mohamed, Ahmed K. Aldhalmi** and **Ayman A. Swelum**: Data curation. **Ahmed K. Aldhalmi, Mohammad M. H. Khan** and **Ayman A. Swelum**: Original draft writing. **Mohamed E. Abd El‐Hack, Elwy A. Ashour, Laila A. Mohamed, Mohamed H. Negm, Mohammad M. H. Khan, Mahmoud Kamal, Aya Rashad, Ahmed K. Aldhalmi** and **Ayman A. Swelum**: Writing–review and editing. All authors read and approved the final manuscript.

## Conflicts of Interest

The authors declare no conflicts of interest.

## Ethics Statement

The Poultry Research Farm, Department of Poultry at the Faculty of Agriculture at Zagazig University in Zagazig, Egypt, conducted this study. The Institutional Animal Care and Use Committee and the Ethics Committee of the Department of Poultry, Faculty of Agriculture, Zagazig University, Zagazig, Egypt, approved all experimental procedures.

## Consent

The authors have nothing to report.

## Data Availability

The data that support the findings of this study are available from the corresponding author upon reasonable request.
